# Subtype-Specific Survival of Young Women with Breast Cancer and Its Interaction with the Germline BRCA Status

**DOI:** 10.3390/cancers16040738

**Published:** 2024-02-10

**Authors:** Anna Maria Hage, Pimrapat Gebert, Jens-Uwe Blohmer, Elham Hedayati, Dorothee Speiser, Maria Margarete Karsten

**Affiliations:** 1 Department of Gynecology with Breast Center, Charité—Universitätsmedizin Berlin, Corporate Member of Freie Universität Berlin and Humboldt Universität zu Berlin, 10117 Berlin, Germany; anna-maria.hage@charite.de (A.M.H.); jens.blohmer@charite.de (J.-U.B.); dorothee.speiser@charite.de (D.S.); 2 Institute of Biometry and Clinical Epidemiology, Charité—Universitätsmedizin Berlin, Corporate Member of Freie Universität Berlin and Humboldt Universität zu Berlin, 10117 Berlin, Germany; pimrapat.gebert@charite.de; 3 Department of Oncology-Pathology, Karolinska Comprehensive Cancer Centre, 17176 Stockholm, Sweden; elham.hedayati@ki.se

**Keywords:** young women with breast cancer, subtype-specific survival, pathogenic germline BRCA variants

## Abstract

**Simple Summary:**

Young women who develop breast cancer before the age of 40 often harbor a pathogenic germline variant in the *BRCA1* or *BRCA2* gene. The type of affected gene is associated with the expression of the clinical subtype, which in turn is associated with prognosis. To assess prognosis in young women with breast cancer, both aspects need to be considered. This retrospective, single-center cohort study describes survival outcomes in interaction with the aforementioned factors. The results indicate better overall survival in young triple-negative patients with pathogenic germline variants in *BRCA1* or *BRCA2* compared to triple-negative breast cancer without pathogenic variants, but not for other clinical subtypes.

**Abstract:**

Data are scarce on the role of pathogenic germline variants in *BRCA1* and *BRCA2* (gBRCAm) in subtype-specific survival in young women who develop breast cancer under the age of 40. This retrospective, real-world cohort study assessed the distant disease-free survival (DDFS) and overall survival (OS) of young women diagnosed with breast cancer between 2008 and 2019 while taking into consideration the interaction of clinical subtypes and the gBRCA status. Among 473 women, HR+/Her2− was the most common subtype (49.0%), followed by TNBC (31.3%), HR+/Her2+ (13.7%), and Her2+/HR− (5.9%). The gBRCA status was known for 319 cases (gBRCAwt (wild-type – without pathogenic variants in *BRCA1* or *BRCA2*): 204, gBRCA1m: 83, gBRCA2m: 31, 1 patient with both). The distribution of clinical subtypes varied depending on the gBRCA status (*p* < 0.001). In survival analysis with a median follow-up of 43 months, the unadjusted DDFS and OS were worse for gBRCAwt TNBC compared to both HR+ subtypes, but not for gBRCAm TNBC patients. T-stage, nodal involvement, and the gBRCA status were identified as significant for survival in TNBC. In TNBC, gBRCAm was associated with better DDFS and OS than gBRCAwt (5-year DDFS 81.4% vs. 54.3%, *p* = 0.012 and 5-year OS 96.7% vs. 62.7%, *p* < 0.001). In contrast, in HR+/Her2− patients, gBRCAm patients showed a tendency for worse survival, though not statistically significant. Subtype-specific survival in young women with breast cancer needs to be evaluated in interaction with the gBRCA status. For TNBC, gBRCAm is of favorable prognostic value for overall survival, while patients with gBRCAwt TNBC need to be considered to have the highest risk for adverse survival outcomes.

## 1. Introduction

Young age at diagnosis used to be considered an independent risk factor for death in patients with breast cancer (BC) [1,2]. However, the prognostic effect of young age has more recently been shown to vary by clinical subtypes, which are defined mostly by the expression of hormone receptors (HR), human epidermal growth factor receptor 2 (Her2), or lack thereof [3,4,5]. Additionally, young women who develop breast cancer under the age of 40 (YWBC) harbor deleterious germline mutations in *BRCA1* (gBRCA1m) or *BRCA2* (gBRCA2m) in approximately 12% of cases [6,7,8].

Since the type of affected gene significantly influences the tumor phenotype [8,9] with higher proportions of triple-negative tumors (TNBC) in gBRCA1m patients and higher proportions of HR+/Her2− tumors in gBRCA2m patients, it is crucial to investigate its prognostic effect in interaction with clinical subtypes when discussing prognosis in YWBC [8,10,11]. Despite that, there is a gap in investigating survival in YWBC in the context of both clinical subtype and the gBRCA status [3,4,12,13,14]. Possible explanations are the small proportion of breast cancer that occurs under the age of 40 (e.g., only 4% in Germany [15] and 4% in the US [16]), incomplete gBRCA testing in YWBC [17,18], and a lack of access to genetic results from routine care for research.

However, YWBC face several age-specific challenges [19,20], including high mortality rates and high proportions of unfavorable tumor characteristics like triple-negative status [4,8]. Lambertini et al. showed that distant disease-free survival (DDFS) and overall survival (OS) in YWBC with gBRCA1m and gBRCA2m are similar when investigated for the same hormone receptor status; therefore, they can be investigated together as gBRCAm for subtype-specific survival [11].

The existing data regarding the effects of gBRCAm have often been inconclusive with reports of better, worse, and similar outcomes in women with gBRCAm early breast cancer, and data are partly still from the 1990s [7]. Some more recent data that discuss the prognostic value of gBRCAm in the context of clinical subtypes indicate a favorable effect of gBRCAm for TNBC during the first 2 years after diagnosis [8]. Also, a reverse association of the prognostic value of gBRCAm with a potential negative effect for HR+ BC is being discussed, but the evidence is still very limited [10,11,21]. In Her2+ disease, young age and gBRCAm both do not seem to impact prognosis [3,4,11,22].

Given the little existing data and changing therapy strategies like the addition of platinum compounds to the chemotherapy of early TNBC, it is necessary to continue the discussion about the prognosis of YWBC and include more recent data.

Hence, in this article, we used recent real-world data to describe characteristics at first diagnosis and investigate distant disease-free and overall survival in YWBC while also taking into consideration the interaction of clinical subtypes with the gBRCA status.

## 2. Materials and Methods

### 2.1. Trial Design and Patient Cohort

This study was conducted in the framework of Berlin Young Patients (BYP), a single-center registry for women with breast cancer under the age of 40 (DRKS00021459). Women diagnosed between 2008 and 2019 and who presented at Charité—Universitaetsmedizin Berlin were included. Their real-world data were collected retrospectively from electronic medical records after obtaining approval from the Charité ethics committee (EA1/153/18). No written informed consent was required as local legal regulations and ethics approval allowed the use of retrospective data from clinical routine.

If patients were diagnosed and/or treated outside of Charité for their first occurrence of breast cancer, data were collected from available records from external breast care institutions. The reporting in this manuscript is compliant with STROBE guidelines [23].

### 2.2. Pathology Findings

We extracted pathology findings from electronic medical records and defined clinical subtypes as follows:

- TN (Triple-negative: hormone receptor-negative and Her2/neu-negative);
- HR+/Her2− (hormone receptor-positive, Her2/neu-negative);
- HR+/Her2+ (hormone receptor-positive, Her2/neu-positive);
- Her2+/HR− (Her2/neu-positive, hormone receptor-negative).

The threshold for hormone receptor positivity (HR+) was set at >10%. “Low-positive” results <10% were categorized as hormone receptor-negative [24,25].

Immunohistological stains of 3+ and/or positive results for gene amplification in in situ hybridization (FISH, CISH, or SISH) were defined for the categorization of Her2/neu as positive.

### 2.3. Germline Testing

Results from germline testing were extracted from medical records and grouped as follows:

- gBRCAm (pathogenic variant in *BRCA1* or *BRCA2*);
- gBRCAwt (wild type, meaning no pathogenic variant in *BRCA1* or *BRCA2*);
- No data (not tested or missing due to testing in an external institution).

In clinical routine, the definition of pathogenic variant is based on the classification of Plon et al. following clinical relevance, with pathogenic variant meaning class IV and class V and wild type meaning class I and II without clinical relevance or class III, variant of unknown significance (VUS) [26].

Patients diagnosed with other cancer susceptibility gene variants (*n* = 14) were excluded because the group was too small and heterogenous to explore their combination with clinical subtypes.

### 2.4. Tumor and Nodal Stage and Clinical Variables

To report the tumor stage and nodal involvement at the time of first diagnosis as accurately as possible, the pathological TNM stage (pTNM) was used primarily. If the patient received neoadjuvant chemotherapy or pTMN was missing, the clinical TNM stage was used instead.

Any initiated chemotherapy with the use of a platinum compound was defined as “platinum-based chemotherapy”.

### 2.5. Survival Analysis and Survival Endpoints

For survival analysis, only patients without primary metastasis and no history of other cancer diseases were included. Survival was calculated from the date of first diagnosis to event or last follow-up. The event of first distant metastasis was used to describe distant disease-free survival (DDFS) and death from any cause for overall survival (OS).

### 2.6. Statistical Analysis

Descriptive analysis of the distribution of clinical subtypes by gBRCA status was performed using the Pearson Chi-square test. Survival outcomes were visualized with Kaplan–Meier curves and compared with log-rank or Breslow’s test. Cox-proportional hazard models were used to explore the association of patient characteristics with DDFS and OS for each subtype. Testing of the proportional-hazards assumption was carried out using the Schoenfeld residuals. Factors with statistical significance in the univariate analysis were included in a multivariable Cox-proportional hazard model. Hazard ratios (HRs) and 95% confidence intervals (CIs) were reported. Statistical testing was carried out within an exploratory framework at a two-sided significance level of α = 0.05 without adjustment for multiple testing. Statistical analyses were performed with IBM SPSS Statistics for Windows, Version 27.0 (Armonk, NY, USA: IBM Corp) and Stata IC15 (StataCorp, 2017, College Station, TX, USA).

## 3. Results

### 3.1. Histopathological and Clinical Features

A total of 473 young breast cancer patients under the age of 40 were included (Supplementary Figure S1) to describe histopathological and clinical features at first diagnosis (Table 1). HR+/Her2− was the most common subtype (49.0%, *n* = 232), followed by TNBC (31.3%, *n* = 148), HR+/Her2+ (13.7%, *n* = 65), and HR−/Her2+ (5.9%, *n* = 28).

**Figure 1:**
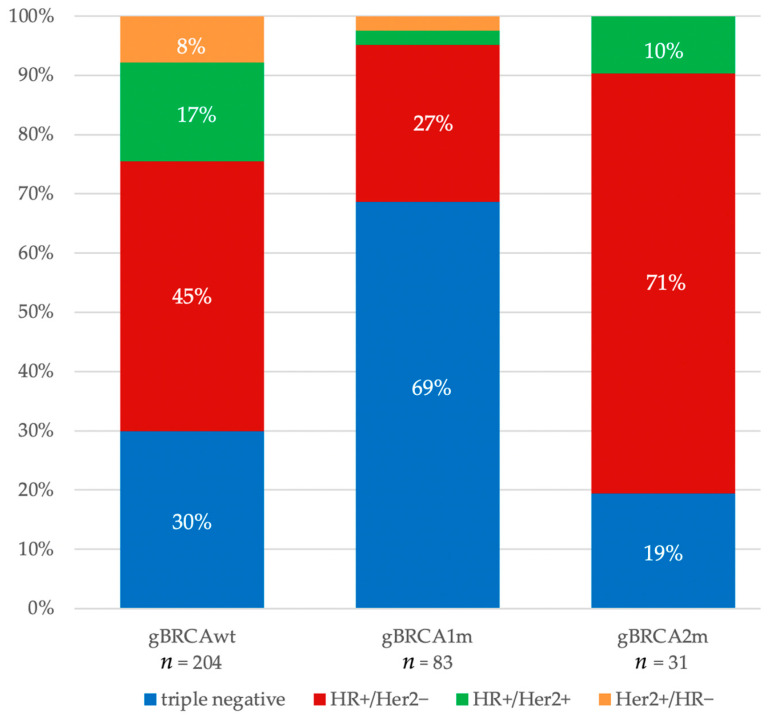
Distribution of clinical subtypes by gBRCA status in baseline study cohort. gBRCAwt: 30% TNBC, 45% HR+/Her2−, 17% HR+/Her2+, 8% Her2+/HR−. gBRCA1m: 69% TNBC, 27% HR+/Her2−, 2% HR+/Her2+, 2% Her2+/HR−. gBRCA2m: 19% TNBC, 71% HR+/Her2−, 10% HR+/Her2+.

However, the distribution of clinical subtypes varied depending on the gBRCA status and the type of affected gene (*BRCA1* vs. *BRCA2*) (*p* < 0.001) (Figure 1). Patients with no available germline testing results (not tested/missing) were older and more likely to suffer from HR+ disease than tested patients (Supplementary Table S1).

### 3.2. Distant Disease-Free Survival and Overall Survival

After excluding patients with primary metastasis or unknown metastatic status at first diagnosis and patients with a history of other cancer diseases, 426 patients were included in the survival analysis.

During a median follow-up time of 43 months (min. 0.1, max. 167), 114 events of distant metastasis (26.7%) as well as 65 events of death (15.2%) were observed.

**Figure 2:**
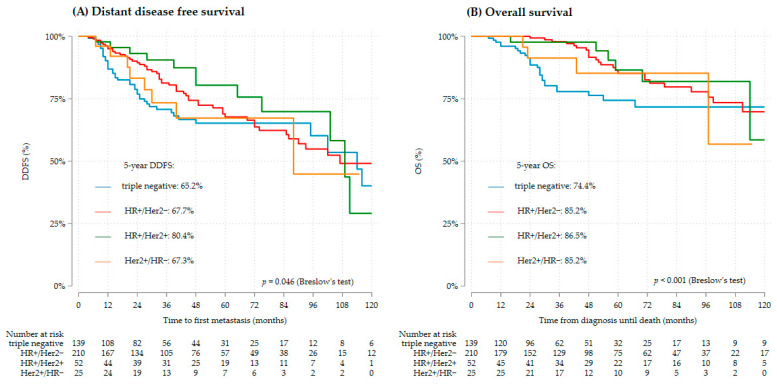
Survival by clinical subtype (*n* = 427), (**A**) DDFS, (**B**) OS.

Overall, TNBC had worse DDFS than both HR+ subgroups (*p* = 0.03 each) (Figure 2A), as well as worse OS (*p* < 0.001 for HR+/Her2−, *p* = 0.02 for HR+/Her2+) (Figure 2B). However, when stratifying for the gBRCA status, this difference remained only for gBRCAwt TNBC. The DDFS and OS of gBRCAm TNBC was not worse compared to the other clinical subtypes (Supplementary Figure S2). Patient characteristics by gBRCA status of the study cohort for survival analysis are provided in Supplementary Table S2.

**Figure 3:**
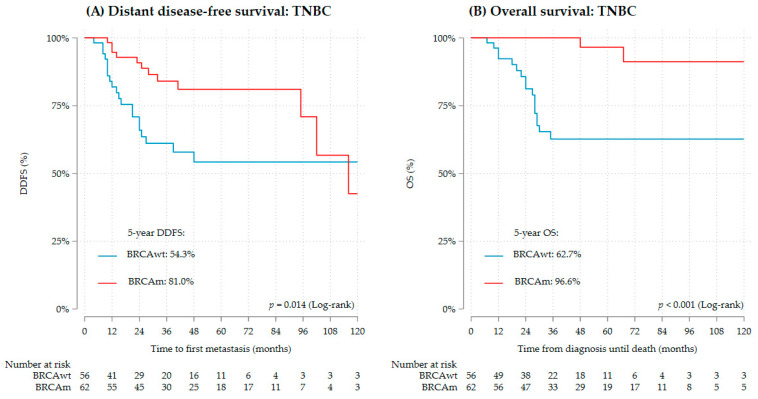
Survival of TNBC by gBRCA status (*n* = 118), (**A**) DDFS, (**B**) OS.

gBRCAwt TNBC was associated with worse DDFS than gBRCAm TNBC (*p* = 0.014), with DDFS at 2 years of 66.0% vs. 90.8% and 54.3% vs. 81.0% at 5 years (Figure 3A). The unadjusted effect had a hazard ratio of 2.39 (95% CI 1.17–4.90). Both T-stage and nodal involvement significantly affected DDFS alongside gBRCAm (Table 2). In a multivariable model with adjustment for the nodal status, the effect of the gBRCA status lost statistical significance but continued with an only mildly attenuated effect size (*p* = 0.057, HR 2.06, 95% CI 0.98–4.34) (Table 3).

Also, OS was worse in gBRCAwt TNBC than in gBRCAm TNBC (*p* < 0.001), with OS at 2 years of 81.1% vs. 100% and 62.7% vs. 96.7% at 5 years (Figure 3B). The unadjusted effect had a hazard ratio of 7.19 (95% CI 2.10–24.61). Both T-stage and nodal involvement significantly affected OS alongside gBRCAm (Table 2). After adjustment for the nodal status, the effect of gBRCAm remained statistically significant and the effect size was mildly attenuated (*p* = 0.004, HR 6.38, 95% CI 1.81–22.49) (Table 3). Adjustment for T-stage led to similar observations (Supplementary Table S3). The proportional hazard assumption was invalid in both models for OS in TNBC and in the multivariate model for DDFS, but all multivariate models gained a valid proportional hazard assumption when all cases were censored at 5 years.

gBRCA1m and gBRCA2m both had better DDFS and OS than gBRCAwt TNBC (Supplementary Figure S3).

There was no significant interaction between the type of chemotherapy regimen (platinum-based vs. other) and gBRCAm TNBC for DDFS and OS (Supplementary Table S4).

**Figure 4:**
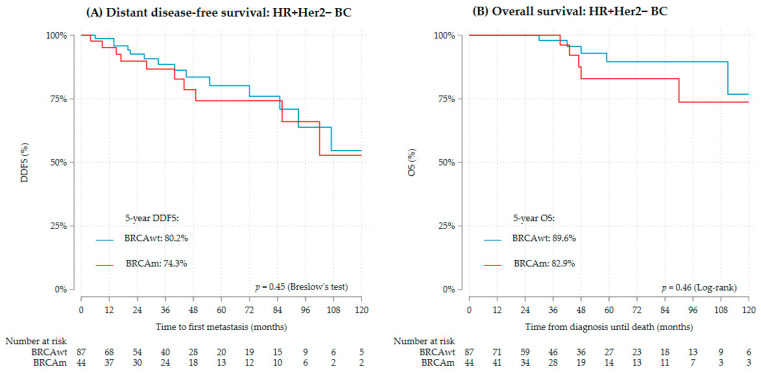
Survival of HR+/Her2− BC by gBRCA status (*n* = 131), (**A**) DDFS, (**B**) OS.

In contrast to that, there was a tendency for better DDFS and OS in gBRCAwt HR+/Her2− than gBRCAm HR+/Her2− (5-year DDFS 80.2% vs. 74.3% and OS 89.6% vs. 82.9%) (Figure 4, Table 4 and Table 5), but this was not statistically significant. Further subgroup analysis showed worse DDFS and OS in gBRCA1m HR+/Her2− than gBRCA2m HR+/Her2−, but this was not statistically significant (Supplementary Figure S4). gBRCA1m HR+/Her2− also had more high-grade disease and tumors with high proliferative indices (Supplementary Table S5).

Also, no statistically significant differences for DDFS and OS were found in the other clinical subtypes when stratifying for the gBRCA status.

## 4. Discussion

In this investigation, the gBRCA status in YWBC affected the distribution of clinical subtypes with higher proportions of TNBC in gBRCA1m patients and higher proportions of HR+/Her2− disease in gBRCA2m patients. Young women with gBRCAwt had high proportions of TNBC and Her2+ disease. TNBC remained the subtype with the worst prognosis among YWBC, as also seen in other studies [3]. However, in the subgroup analysis, gBRCAm TNBC had better DDFS and OS than gBRCAwt TNBC. It is likely that some patients with gBRCAm who were tested predictively, were diagnosed in earlier disease stages in breast cancer screening. Nevertheless, this tendency for better survival also remained after adjustment for T-stage and nodal involvement, yet the effect of gBRCAm remained statistically significant only for OS.

We detected an early advantage in survival of gBRCAm TNBC, as also described in the POSH study [8], that so far is the largest prospective study that compared the BC survival of young women by gBRCA status across clinical subtypes. Copson et al. described a favorable effect of gBRCAm on OS in young women with TNBC with only an early advantage at 2 years, but not at 5 and 10 years, compared to gBRCAwt TNBC [8]. Meanwhile, our data indicate an advantage for gBRCAm TNBC also at 5 years in DDFS and OS. The POSH study recruited patients between 2000 and 2008—since then, treatment strategies have been optimized and platinum-based chemotherapy has been introduced to the routine care of triple-negative early breast cancer.

Young age, gBRCAm, and triple-negative subtype all have been shown to be associated with high chemosensitivity and should be considered a key factor for the discussion of survival outcomes. TNBC shows the highest chemosensitivity among YWBC with 39% showing pathological complete response (pCR) after neoadjuvant anthracycline or taxane-based chemotherapy (compared to 29% for Her2+/HR−, 19% for HR+/Her2+, and 11% for HR+/Her2−) [12]. The recent addition of platinum compounds to neoadjuvant chemotherapy regimens has increased pCR rates in young women with TNBC even more and has potentially also improved survival [14]. gBRCAm is also associated with high chemosensitivity since it leads to a deficiency in DNA repair, so cytotoxic therapies inducing DNA damage might be especially effective [27]. Therefore, a positive value of gBRCAm on therapy response and survival in TNBC patients is being discussed [8,21,28,29]. Studies, yet not exclusively on young women, report higher response rates to several cytotoxic therapies in gBRCAm than gBRCAwt TNBC [28,29,30]. 

While the addition of platinum-based compounds to the chemotherapy of TNBC has been shown to be associated with further improved pCR rates in general [14,31,32], its interaction with gBRCAm and its long-term survival benefit for young women with triple-negative early breast cancer is not entirely clear. In our small cohort, we could not find an interaction between the use of platinum compounds and gBRCAm for OS or DDFS. Other existing data show conflicting results: In the TNT trial [28], the treatment response in advanced TNBC was reported to be better with platinum-based chemotherapy than with doxycycline-based chemotherapy for gBRCAm patients. Yet, a smaller overall response rate and no difference between the two regimens was observed in gBRCAwt TNBC [28]. In contrast, the INFORM trial found a better response to neoadjuvant doxorubicin–cyclophosphamide (AC) than single-agent cisplatin in gBRCAm TNBC patients [33]. Furthermore, analyses of the GeparSixto trial [29] and the BrighTNess trial [34] reported an increased pCR rate in gBRCAwt TNBC patients but not in gBRCAm TNBC after the addition of platinum-based agents to neoadjuvant chemotherapy in early breast cancer. Therefore, it has been hypothesized that gBRCAm tumors may already be intrinsically highly sensitive to anthracycline–taxane chemotherapy and possibly benefit less from additional platinum compounds [34]. This issue is particularly important to further evaluate in young women not only in terms of survival but also in the context of quality of life and long-term side effects since platinum compounds add toxicity to the therapy and YWBC face a longer survivorship period than older women due to their age.

Considering the differences in response to chemotherapeutic treatment and in survival outcomes, gBRCAm TNBC and gBRCAwt TNBC should be investigated separately, ultimately considering them two different clinical subtypes. 

Thus, also the discussion about the impact of young age on the prognosis of breast cancer needs to be continued. A recent SEER-data analysis found that young age remains an independent risk for worse outcomes only for low-grade HR+ disease, but not for other clinical subtypes [3]. Further evaluation in the context of gBRCAm in TNBC could therefore complement this discussion. 

To potentially replicate the findings in our study (5-year DDFS with 54% in gBRCAwt and 81% in gBRCAm TNBC) with an HR = 2, a minimum sample size of 53 cases per group is required to achieve a power of 80%, and 67 cases per group for a power of 90%.

In contrast to TNBC, gBRCAm HR+/Her2− disease had a trend for worse survival compared to gBRCAwt HR+/Her2−, but this was not statistically significant. Nevertheless, these findings are worth mentioning since the association with gBRCAm goes in the opposite direction to TNBC. Hence, in statistical analysis, the gBRCA status and clinical subtypes need to be considered as an interaction and simple adjustment for hormone recptor status might mask this reverse association.

Some recent studies have also discussed a reverse association of the prognostic value of gBRCAm for TNBC and HR+ BC [10,11,21]. The negative effect of gBRCAm in HR+ tumors is still unclear but is speculated that it could be associated with younger age at the time of diagnosis [8]. Younger women face a longer premenopausal period and therefore more hormonal exposure during survivorship than gBRCAwt. Additionally, young age is associated with decreased adherence to antihormonal therapy [35]. Accordingly, future studies should include data on menopausal status and adherence to endocrine therapy to further investigate survival of young women with HR+/Her2− BC.

Due to the relatively low incidence of breast cancer in women under the age of 40, our study cohort is small compared to other breast cancer analyses, and the interpretation of the results in the small subgroup analyses is limited. The hazard ratios of the Cox regression models need to be interpreted with care since the proportional hazard assumptions were not met due to the small event numbers. Yet, regarding the specificity of this patient population, this study shows one of the largest real-world data collections that includes the gBRCA status together with histopathological and clinical data and follow-up for young women with early breast cancer in Germany.

In the sight of emerging BRCA-specific therapies like PARP Inhibitors in the adjuvant setting, data on the interaction of gBRCAm and subtype-specific survival assist the discussion of study results and new treatment approaches. The OlympiA trial [36] already indicates potential survival benefits in early-stage gBRCAm HR+ and TNBC after the use of the PARP Inhibitor Olaparib [37].

The study design as a retrospective cohort study is a limitation resulting in not always fully complete clinical data. Yet, documentation for this study was performed by one medical doctor with the same documentation protocol for all cases to ensure high data quality. Charité—Universitaetsmedizin Berlin is a university hospital with a specialized center for hereditary breast and ovarian cancer. Hence, we expect referral bias with an over-representation of advanced-stage breast cancer cases as well as of patients with pathogenic germline variants, and we do not draw conclusions regarding prevalence. For 154 patients (32,6%), there was no information about the gBRCA status available. In Germany, not all YWBC qualify for germline testing. German guidelines allow germline testing in young women who develop breast cancer after their 36th birthday only under specific conditions [38]. Since we observed the highest quota of missing values for the gBRCA status in YWBC diagnosed between 36 and 39 years of age, we assume this guideline contributed to patients in this age group being tested less frequently. Furthermore, the German Genetic Diagnostics Law (“Gendiagnostikgesetz”) puts genetic data under special protection, meaning that germline testing results are not routinely included in patients’ general medical records and are therefore not always accessible. Moreover, the clinical subtypes defined in this study can be further differentiated by grading and/or proliferation rate (e.g., Luminal A/B), and gBRCAm can be further differentiated in gBRCA1m and gBRCA2m.

## 5. Conclusions

In summary, researching YWBC is impeded by the low incidence of breast cancer under the age of 40 and the high heterogeneity of the disease. Therefore, real-world data from registries are important to discuss current prognostic differences between clinical subtypes, especially with changing therapy strategies. 

Subtype-specific survival in young women with breast cancer needs to be evaluated in interaction with the gBRCA status. For TNBC, gBRCAm is of favorable prognostic value for overall survival, while patients with gBRCAwt TNBC need to be considered to have the highest risk for adverse survival outcomes. Our data call for confirmation and comparison in bigger cohorts. In the framework of precision medicine, one should consider redefining clinical subtypes in young women by splitting the group of TNBC by the gBRCA status. Therapeutic strategies for young women with gBRCAwt TNBC and gBRCAm TNBC need to be further and separately evaluated in prospective, randomized controlled trials, keeping in mind the inherent differences in chemosensitivity and prognosis.

## Figures and Tables

**Table 1:** Patient characteristics (study cohort for baseline characteristics).

Patient Characteristics *		Total—*n* (%) (*n* = 473)		Clinical Subtype—*n* (%)							
				Triple Negative (*n* = 148, 31.3%)		HR+/Her2− (*n* = 232, 49.0%)		HR+/Her2+ (*n* = 65, 13.7%)		Her2+/HR− (*n* = 28, 5.9%)	
age at initial diagnosis	≤25	14	(3.0)	4	(2.7)	6	(2.6)	4	(6.2)	0	(0.0)
	26–30	70	(14.8)	29	(19.6)	27	(11.6)	9	(13.8)	5	(17.9)
	31–35	184	(38.8)	71	(48.0)	78	(33.5)	24	(36.9)	11	(39.3)
	36–39	206	(43.5)	44	(29.7)	122	(52.4)	28	(43.1)	12	(42.9)
median age (range)		35	(22–39)	34	(22–39)	36	(22–39)	35	(24–39)	35	(26–39)
germline BRCA status	gBRCAwt	204	(43.1)	61	(41.2)	93	(40.1)	34	(52.3)	16	(57.1)
	gBRCA1m	83	(17.5)	57	(38.5)	22	(9.5)	2	(3.1)	2	(7.1)
	gBRCA2m	31	(6.6)	6	(4.1)	22	(9.5)	3	(4.6)	0	(0.0)
	gBRCA1m and gBRCA2m	1	(0.2)	1	(0.7)	0	(0.0)	0	(0.0)	0	(0.0)
	not tested/missing	154	(32.6)	23	(15.5)	95	(40.9)	26	(40.0)	10	(35.7)
histopathology	NST/invasive ductal	392	(87.9)	125	(88.7)	185	(84.9)	57	(93.4)	25	(96.2)
	lobular	23	(5.1)	1	(0.7)	19	(8.7)	3	(4.9)	0	(0.0)
	medullary	11	(2.5)	9	(6.4)	2	(0.9)	0	(0.0)	0	(0.0)
	mucinous	7	(1.6)	0	(0.0)	7	(3.2)	0	(0.0)	0	(0.0)
	metaplastic	7	(1.6)	6	(4.3)	1	(0.5)	0	(0.0)	0	(0.0)
	other	6	(1.3)	0	(0.0)	4	(1.8)	1	(1.6)	1	(3.8)
	missing	27		7		14		4		2	
T stage	T1	169	(37.7)	45	(32.1)	91	(41.0)	26	(44.1)	7	(25.9)
	T2	207	(46.2)	75	(53.6)	95	(42.8)	25	(42.4)	12	(44.4)
	T3	54	(12.1)	15	(10.7)	28	(12.6)	6	(10.2)	5	(18.5)
	T4	18	(4.0)	5	(3.6)	8	(3.6)	2	(3.4)	3	(11.1)
	missing	25		8		10		6		1	
nodal involvement	N0	222	(49.6)	76	(54.7)	102	(46.2)	36	(59.0)	8	(29.6)
	N+	226	(50.4)	63	(45.3)	119	(53.8)	25	(41.0)	19	(70.4)
	missing	25		9		11		4		1	
primary metastasis	M0	433	(92.5)	143	(97.3)	212	(91.8)	53	(85.5)	25	(89.3)
	M1	35	(7.5)	4	(2.7)	19	(8.2)	9	(14.5)	3	(10.7)
	missing	5		1		1		3		0	
tumor grading	G1	23	(5.2)	0	(0.0)	20	(90)	3	(5.3)	0	(0.0)
	G2	179	(40.3)	21	(15.1)	123	(55.2)	27	(47.4)	8	(32.0)
	G3	242	(54.5)	118	(84.9)	80	(35.9)	27	(47.4)	17	(68.0)
	missing	29		9		9		8		3	
Ki67 proliferative index	low (≤15%)	105	(24.4)	3	(2.3)	84	(38.9)	14	(23.0)	4	(18.2)
	intermediate	138	(32.0)	28	(21.2)	76	(35.2)	27	(44.3)	7	(31.8)
	high (>35%)	188	(43.6)	101	(76.5)	56	(25.9)	20	(32.8)	11	(50.0)
	missing	42		16		16		4		6	
chemotherapy treatment of first occurrence	no	70	(15.0)	7	(4.8)	60	(26.2)	3	(4.8)	0	(0.0)
	yes	397	(85.0)	140	(95.2)	169	(73.8)	60	(95.2)	28	(100.0)
	missing	6		1		3		2		0	
use of platinum com-pounds in chemotherapy of first occurrence	no (other chemo)	309	(81.3)	84	(61.8)	157	(97.5)	49	(87.5)	19	(70.4)
	yes	71	(18.7)	52	(38.2)	4	(2.5)	7	(12.5)	8	(29.6)
	missing	17		4		8		4		1	

* Percentages may not total 100 because of rounding.

**Table 2:** Univariate Cox proportional hazard models for DDFS and OS in TNBC.

Factor	Levels	DDFS		OS	
		HR (95% CI)	*p*-Value	HR (95% CI)	*p*-Value
age		1.04 (0.96, 1.14)	0.332	1.08 (0.97, 1.20)	0.173
germline BRCA status	gBRCAm	1.00		1.00	
	gBRCAwt	2.39 (1.17, 4.90)	0.017	7.19 (2.10, 24.61)	0.002
T stage	T1	1.00	0.004	1.00	0.071
	T2	5.49 (1.66, 18.19)	0.005	3.76 (1.10, 12.87)	0.035
	T3/T4	9.26 (2.45, 35.00)	0.001	5.11 (1.14, 23.00)	0.033
nodal involvement	N0	1.00		1.00	
	N+	3.71 (1.79, 7.69)	<0.001	3.90 (1.54, 9.86)	0.004
tumor grading	G1/G2	1.00		1.00	
	G3	0.76 (0.33, 1.74)	0.514	1.42 (0.43, 4.75)	0.566
Ki67 proliferative	low/intermediate (≤35%)	1.00		1.00	
index	high (>35%)	2.29 (0.95, 5.53)	0.066	2.06 (0.74, 5.69)	0.166
platinum-based	yes	1.00		1.00	
chemotherapy	other chemo	1.71 (0.89, 3.31)	0.109	1.12 (0.48, 2.60)	0.793

**Table 3:** Multivariate Cox proportional hazard model for DDFS and OS in TNBC.

Factor	Levels	DDFS		OS	
		HR (95% CI)	*p*-Value	HR (95% CI)	*p*-Value
germline BRCA status	gBRCAm	1.00		1.00	
	gBRCAwt	2.06 (0.98, 4.34)	0.057	6.38 (1.81, 22.49)	0.004
nodal status	N0	1.00		1.00	
	N+	5.03 (2.03, 12.44)	<0.001	6.42 (1.82, 22.60)	0.004

**Table 4:** Univariate Cox proportional hazard models for DDFS and OS in HR+/Her2−.

Factor	Levels	DDFS		OS	
		HR (95% CI)	*p*-Value	HR (95% CI)	*p*-Value
age		0.98 (0.92, 1.05)	0.630	0.96 (0.88, 1.04)	0.291
germline BRCA status	gBRCAwt	1.00		1.00	
	gBRCAm	1.26 (0.56, 2.85)	0.572	1.55 (0.48, 5.13)	0.464
T stage	T1	1.00	0.130	1.00	0.408
	T2	1.29 (0.67, 2.49)	0.438	1.28 (0.51, 3.20)	0.598
	T3/T4	2.32 (1.02, 5.26)	0.044	2.14 (0.70, 6.56)	0.182
nodal involvement	N0	1.00		1.00	
	N+	1.27 (0.70, 2.29)	0.431	1.59 (0.66, 3.83)	0.306
tumor grading	G1/G2	1.00		1.00	
	G3	1.96 (1.11, 3.46)	0.020	3.14 (1.34, 7.34)	0.008
Ki67 proliferative	low/intermediate (≤35%)	1.00		1.00	
index	high (>35%)	1.18 (0.60, 2.29)	0.635	0.95 (0.35, 2.59)	0.917
chemotherapy	no chemo	1.00		1.00	
	yes	1.25 (0.64, 2.43)	0.513	1.78 (0.61, 5.17)	0.291

**Table 5:** Multivariate Cox proportional hazard model for DDFS and OS in HR+/Her2−.

Factor	Levels	DDFS		OS	
		HR (95% CI)	*p*-Value	HR (95% CI)	*p*-Value
germline BRCA status	gBRCAwt	1.00		1.00	
	gBRCAm	1.71 (0.73, 4.00)	0.217	1.66 (0.50, 5.53)	0.411
T stage	T1	1.00			
	T2	1.02 (0.40, 2.61)	0.969	-	-
	T3/T4	3.58 (1.04, 12.27)	0.042		
tumor grading	G1/G2	1.00		1.00	
	G3	2.37 (0.99, 5.71)	0.054	1.74 (0.53, 5.75)	0.368

## Data Availability

Access can be requested from the host institution Charité—Universitaetsmedizin Berlin via the corresponding author. Requests need to be petitioned to and assessed by the Charité Ethics Committee to evaluate concordance with legal and ethical implications and by the research team to evaluate its feasibility.

## Supplementary Materials

The following supporting information can be downloaded at: https://www.mdpi.com/article/10.3390/cancers16040738/s1, Figure S1: Study flow-chart; Figure S2: Survival by clinical subtypes with gBRCAm/gBRCAwt TNBC, A: DDFS, B: OS; Figure S3: Survival of TNBC by gBRCA1m, gBRCA2m and gBRCAwt, A: DDFS, B: OS; Figure S4: Survival of HR+/Her2- BC by gBRCA1m, gBRCA2m and gBRCAwt, A: DDFS, B: OS; Table S1: Patient characteristics by gBRCA testing; Table S2: Patient characteristics by BRCA status.; Table S3: Additional multivariate Cox proportional hazard model for DDFS and OS in TNBC; Table S4: Additional univariate Cox proportional hazard models for DDFS and OS in TNBC; Table S5: Patient characteristics by gBRCA1m, gBRCA2m and gBRCAwt.
