# Supplementary material for: Subtype-Specific Survival of Young Women with Breast Cancer and Its Interaction with the Germline BRCA Status
Source: Cancers (Basel). 2024 Feb 10;16(4):738. doi: 10.3390/cancers16040738 (PMC10887122; doi:10.3390/cancers16040738)
Supplement: Supplementary file 1 [file cancers-16-00738-s001.zip › 2024_02_09_supplementary_subtype_manuscript_cancers_clean.pdf]

Supplementary

Figure S1: Study flow-chart

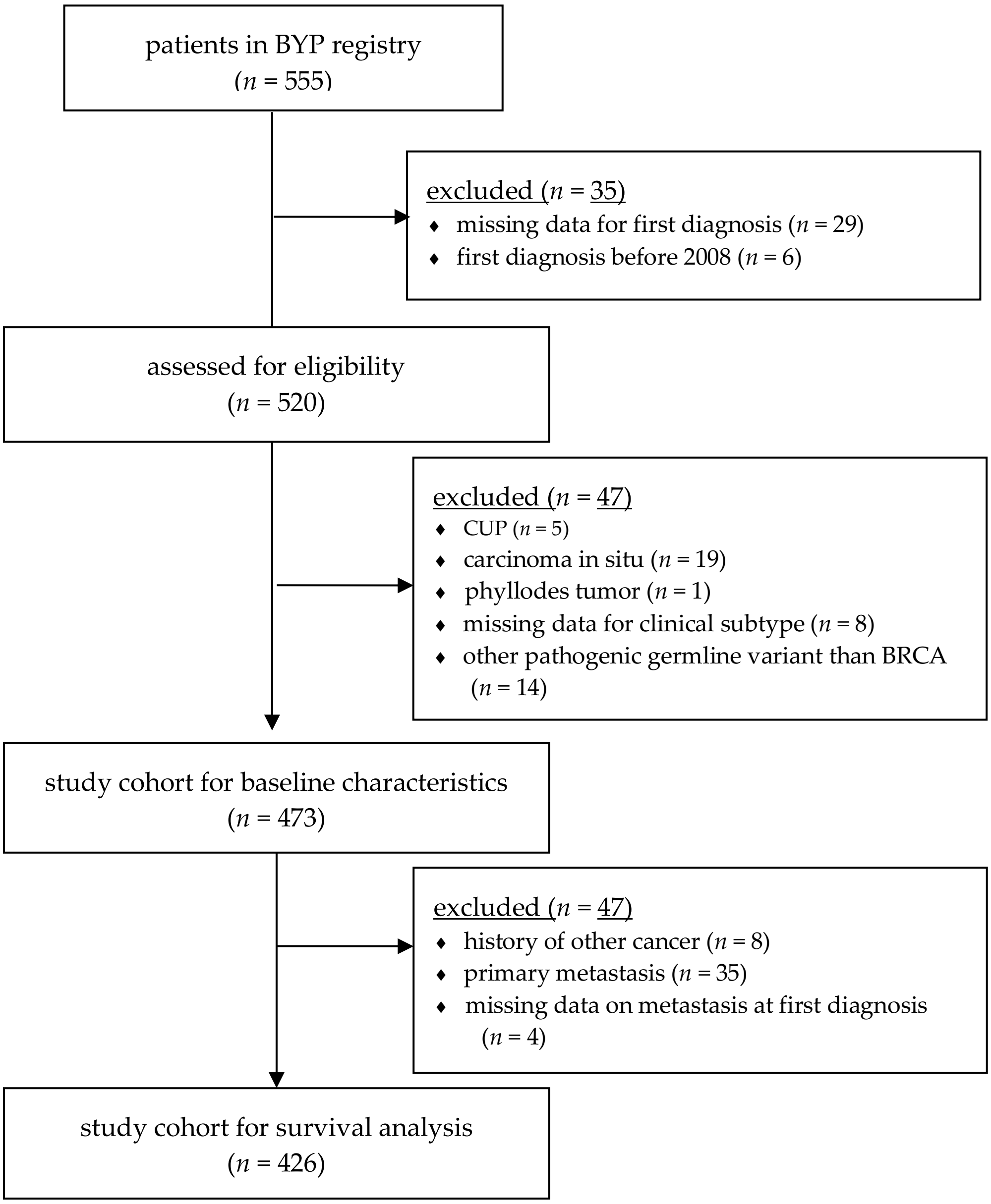

Table S1: Patient characteristics by gBRCA testing (study cohort for baseline characteristics).

		gBRCA testing – *n* (%)			
		tested (*n* = 319)		not tested/missing (*n* = 154)	
age at initial diagnosis	≤25	11	3.4%	3	1.9%
	26-30	52	16.3%	18	11.7%
	31-35	146	45.8%	38	24.7%
	36-39	110	34.5%	95	61.7%
median age (range)		34	(22-39)	36	(24-39)
clinical subtype	triple negative	125	39.2%	23	14.9%
	HR+/Her2−	137	42.9%	95	61.7%
	HR+/Her2+	39	12.2%	26	16.9%
	Her2+/HR−	18	5.6%	10	6.5%
year of first diagnosis	2008	14		22	
	2009	7		12	
	2010	12		25	
	2011	17		18	
	2012	17		15	
	2013	22		13	
	2014	31		8	
	2015	52		7	
	2016	36		9	
	2017	43		7	
	2018	27		6	
	2019	41		12	

Figure S2: Survival by clinical subtype with gBRCAm / gBRCAwt TNBC, (A) DDFS, (B) OS.

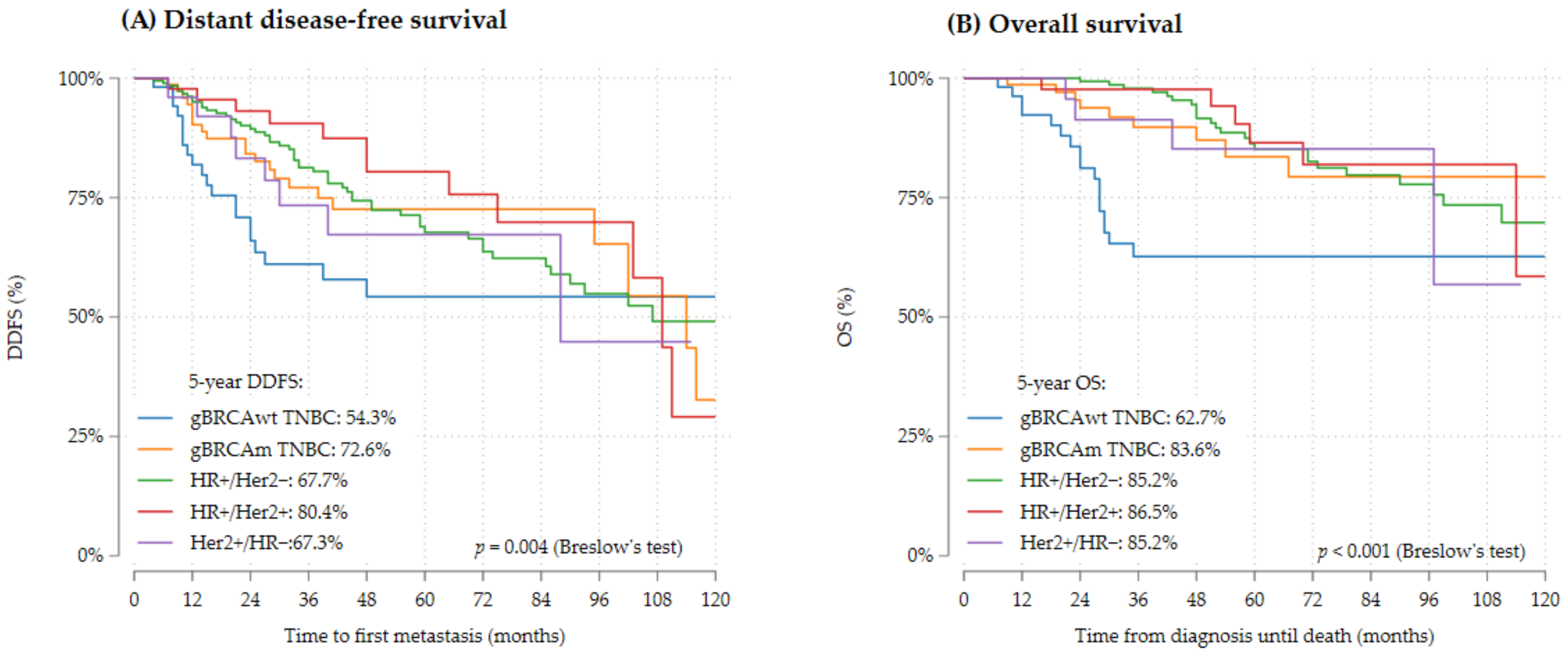

(A) Number at risk

Number at risk	0	12	24	36	48	60	72	84	96	108	120
gBRCAwt TNBC	56	41	29	20	16	11	6	4	3	3	3
gBRCAm TNBC	83	67	53	36	28	20	19	13	9	5	3
HR+/Her2−	210	167	134	105	76	57	49	38	26	15	12
HR+/Her2+	52	44	39	31	25	19	13	11	7	4	1
Her2+/HR−	25	24	19	13	9	7	6	3	2	2	0

(B) Number at risk

Number at risk	0	12	24	36	48	60	72	84	96	108	120
gBRCAwt TNBC	56	49	38	22	18	11	6	4	3	3	3
gBRCAm TNBC	83	71	58	40	33	21	19	13	10	6	6
HR+/Her2−	210	179	152	129	98	75	62	47	37	22	17
HR+/Her2+	52	45	41	34	29	22	17	16	10	8	5
Her2+/HR−	25	25	21	17	12	10	9	5	3	2	0

Table S2: Patient characteristics by BRCA status (study cohort for survival analysis).

		**clinical subtype – *n* (%)**															
		**triple-negative**				**HR+/Her2−**				**HR+/Her2+**				**Her2+/HR−**			
		**gBRCAwt (*n* = 56)**		**gBRCAm (*n* = 62)**		**gBRCAwt (*n* = 87)**		**gBRCAm (*n* = 44)**		**gBRCAwt (*n* = 31)**		**gBRCAm (*n* = 5)**		**gBRCAwt (*n* = 14)**		**gBRCAm (*n* = 2)**	
age at initial diagnosis	≤25	1	1.8%	3	4.8%	2	2.3%	3	6.8%	1	4.0%	1	20.0%	0	0.0%	0	0.0%
	26-30	8	14.3%	15	24.2%	11	12.6%	6	13.6%	2	0.0%	1	20.0%	4	28.6%	0	0.0%
	31-35	26	46.4%	33	53.2%	36	41.4%	22	50.0%	11	40.0%	1	20.0%	6	42.9%	1	50.0%
	36-39	21	37.5%	11	17.7%	38	43.7%	13	29.5%	17	56.0%	2	40.0%	4	28.6%	1	50.0%
median age (range)		34	(25-39)	32	(22-39)	35	(22-39)	34	(23-39)	36	(25-39)	34	(25-37)	32	(26-39)	37	(35-38)
gBRCA status	gBRCA1m	-		55	88.7%	-		22	50.0%	-		2	40.0%	-		2	100.0%
	gBRCA2m	-		6	9.7%	-		22	50.0%	-		3	60.0%	-		0	0.0%
	both	-		1	1.6%	-		0	0.0%	-		0	0.0%	-		0	0.0%
histopathology	NST/ invasive ductal	46	83.6%	55	94.8%	70	84.3%	41	95.3%	30	96.8%	5	100.0%	13	100.0%	2	100.0%
	invasive lobular	1	1.8%	0	0.0%	7	8.4%	1	2.3%	1	3.2%	0	0.0%	0	0.0%	0	0.0%
	medullary	5	9.1%	2	3.4%	1	1.5%	1	2.3%	0	0.0%	0	0.0%	0	0.0%	0	0.0%
	mucinous	0	0.0%	0	0.0%	3	2.9%	0	0.0%	0	0.0%	0	0.0%	0	0.0%	0	0.0%
	metaplastic	3	5.5%	1	1.7%	0	0.0%	0	0.0%	0	0.0%	0	0.0%	0	0.0%	0	0.0%
	other	0	0.0%	0	0.0%	2	2.9%	0	0.0%	0	0.0%	0	0.0%	0	0.0%	0	0.0%
	missing	1		4		4		1		0		0		1		0	
T stage	T1	16	30.8%	24	40.7%	37	43.0%	25	58.1%	15	48.4%	4	80.0%	3	23.1%	1	50.0%
	T2	30	57.7%	30	50.8%	36	41.9%	16	37.2%	15	48.4%	1	20.0%	5	38.5%	1	50.0%
	T3	4	7.7%	3	5.1%	12	14.0%	1	2.3%	1	3.2%	0	0.0%	3	23.1%	0	0.0%
	T4	2	3.8%	2	3.4%	1	1.2%	1	2.3%	0	0.0%	0	0.0%	2	15.4%	0	0.0%
	missing	4		3		1		1		0		0		1		0	
nodal involvement	N0	26	50.0%	34	58.6%	38	43.7%	28	68.3%	23	74.2%	3	60.0%	3	23.1%	1	50.0%
	N+	26	50.0%	24	41.4%	49	56.3%	13	31.7%	8	25.8%	2	40.0%	10	76.9%	1	50.0%
	missing	4		4		0		3		0		0		1		0	
tumor grading	G1	0	0.0%	0	0.0%	8	9.2%	3	6.8%	3	10.3%	0	0.0%	0	0.0%	0	0.0%
	G2	5	9.3%	11	19.6%	48	55.2%	23	52.3%	15	51.7%	3	60.0%	6	46.2%	2	100.0%
	G3	49	90.7%	45	80.4%	31	35.6%	18	40.9%	11	37.9%	2	40.0%	7	53.8%	0	0.0%
	missing	2		6		0		0		2		0		1		0	

Ki67 proliferative	low (≤15%)	1	2.0%	0	0.0%	30	36.6%	12	28.6%	9	29.0%	0	0.0%	3	23.1%	0	0.0%
index	intermediate	8	15.7%	14	25.9%	35	42.7%	11	26.2%	12	38.7%	3	60.0%	4	30.8%	1	50.0%
	high (>35%)	42	82.4%	40	74.1%	17	20.7%	19	45.2%	10	32.3%	2	40.0%	6	46.2%	1	50.0%
	missing	5		8		5		2		0		0		1		0	
chemotherapy	no	0	0.0%	3	4.8%	18	20.9%	13	29.5%	2	6.5%	0	0.0%	0	0.0%	0	0.0%
treatment of first	yes	56	100.0%	59	95.2%	68	79.1%	31	70.5%	29	93.5%	5	100.0%	14	100.0%	2	100.0%
occurrence	missing	0		0		1		0		0		0		0		0	
use of platinum	no (other chemo)	37	67.3%	26	46.4%	66	100.0%	25	86.2%	25	86.2%	5	100.0%	7	50.0%	2	100.0%
compounds in	yes	18	32.7%	30	53.6%	0	0.0%	4	13.8%	4	13.8%	0	0.0%	7	50.0%	0	0.0%
chemotherapy of first	n/a - no chemo	0		3		18		13		2		0		0		0	
occurrence	missing	1		3		3		2		0		0		0		0	

*Percentages may not total 100 because of rounding

Table S3: Additional multivariate Cox proportional hazard model for DDFS and OS in TNBC

factor	levels	DDFS		OS	
		**HR (95% CI)**	***p*-value**	**HR (95% CI)**	***p*-value**
germline BRCA status	gBRCAm	1.00		1.00	
	gBRCAwt	1.81 (0.85, 3.86)	0.125	6.47 (1.84, 22.73)	0.004
T stage	T1	1.00		1.00	
	T2	6.60 (1.54, 28.16)	0.011	4.69 (1.05, 21.02)	0.043
	T3/T4	8.72 (1.67, 45.50)	0.010	4.17 (0.57, 30.25)	0.158

Table S4: Additional univariate Cox proportional hazard models for DDFS and OS in TNBC

factor	levels	DDFS		OS	
		**HR (95% CI)**	***p*-value**	**HR (95% CI)**	***p*-value**
interaction of gBRCAm and chemotherapy	gBRCAm + other chemo	1.00		1.00	
	gBRCAm + platinum	2.18 (0.53, 8.98)	0.280	0.024 (0.00, 4126.22)	0.544
interaction of gBRCAwt and chemotherapy	gBRCAwt + other chemo	1.00		1.00	
	gBRCAwt + platinum	4.34 (1.77, 10.66)	0.001	2.48 (0.95, 6.45)	0.062

Figure S3: Survival of TNBC by gBRCA1m, gBRCA2m and gBRCAwt, (A) DDFS, (B) OS.*

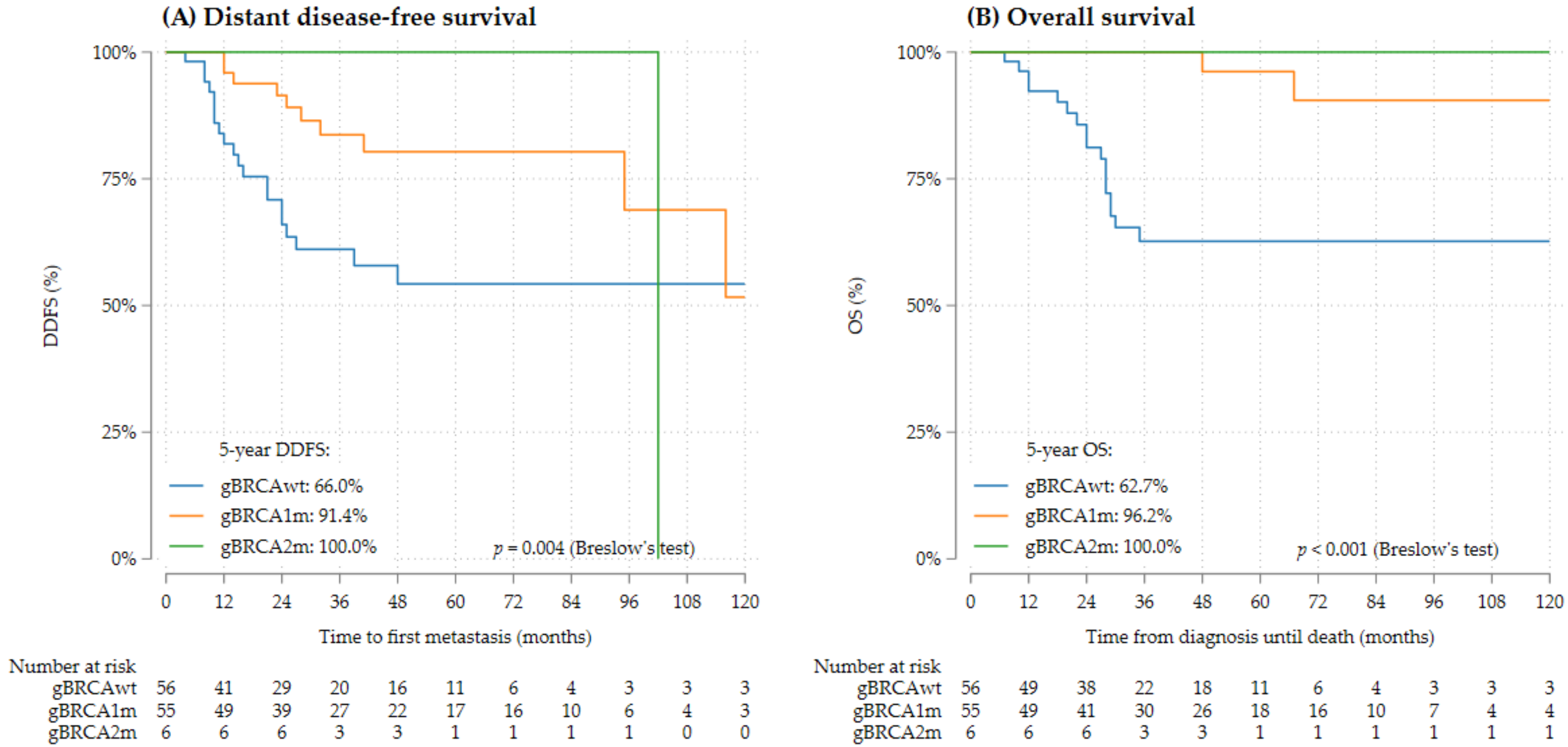

*the patient with both gBRCA1m and gBRCA2m was excluded from this overview

Figure S4: Survival of HR+/Her2− BC by gBRCA1m, gBRCA2m and gBRCAwt, (A) DDFS, (B) OS.

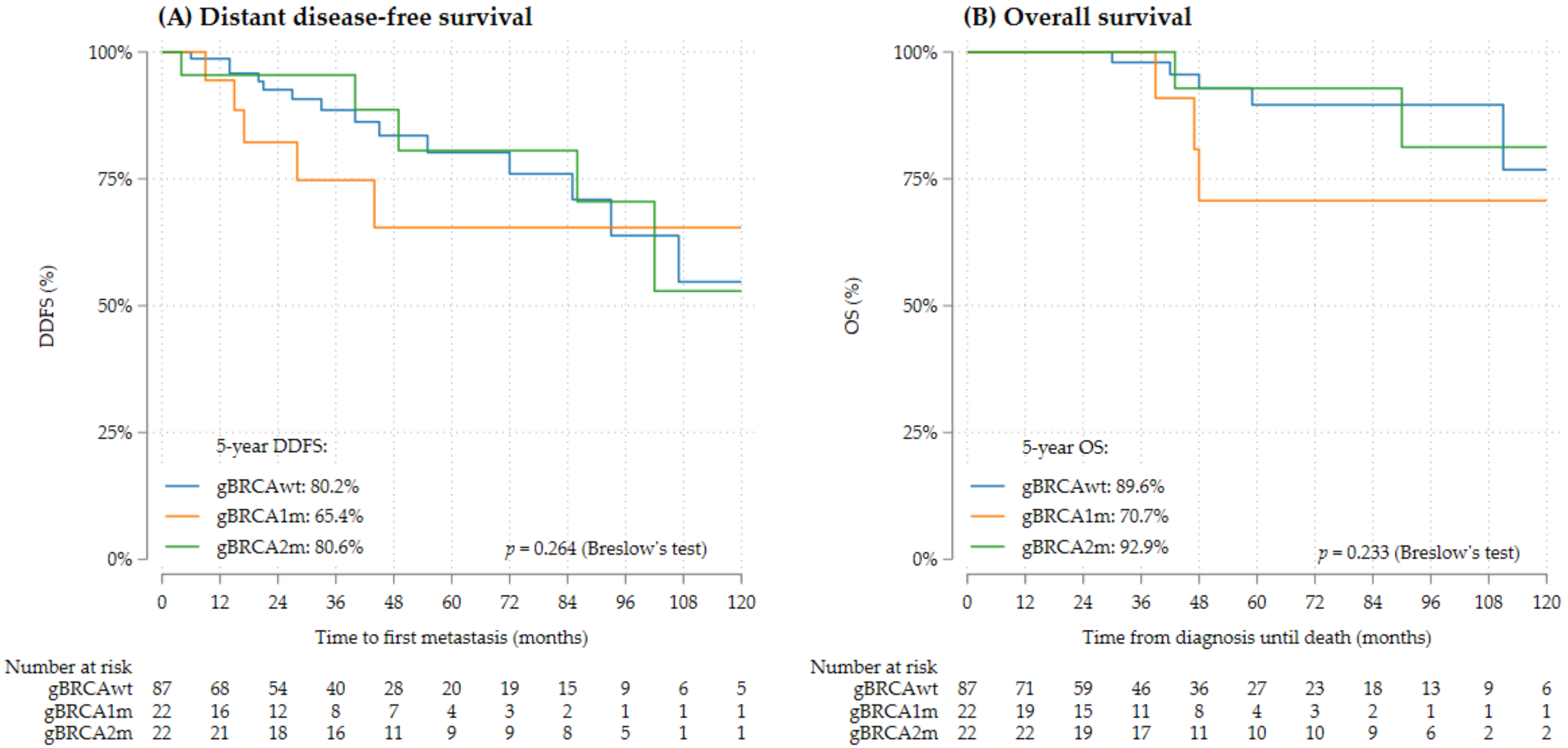

Table S5: Patient characteristics by gBRCA1m, gBRCA2m and gBRCAwt (study cohort for survival analysis).*

		**clinical subtype – *n* (%)**											
		**triple-negative**						**HR+/Her2–**					
		**gBRCAwt (*n* = 56)**		**gBRCA1m (*n* = 55)**		**gBRCA2m (*n* = 6)**		**gBRCAwt (*n* = 87)**		**gBRCA1m (*n* = 22)**		**gBRCA2m (*n* = 22)**	
age at initial diagnosis	≤25	1	1.8%	3	5.5%	0	0.0%	2	2.3%	2	9.1%	1	4.5%
	26-30	8	14.3%	13	23.6%	2	33.3%	11	12.6%	4	18.2%	2	9.1%
	31-35	26	46.4%	30	54.5%	2	33.3%	36	41.4%	12	54.5%	10	45.5%
	36-39	21	37.5%	9	16.4%	2	33.3%	38	43.7%	4	18.2%	9	40.9%
median age (range)		34	(25-39)	32	(22-39)	34	(29-39)	35	(22-39)	34	(24-39)	35	(23-39)
histopathology	NST/ invasive ductal	46	83.6%	48	94.1%	6	100.0%	70	84.3%	21	95.5%	20	95.2%
	invasive lobular	1	1.8%	0	0.0%	0	0.0%	7	8.4%	0	0.0%	1	4.8%
	medullary	5	9.1%	2	3.9%	0	0.0%	1	1.5%	1	4.8%	0	0.0%
	mucinous	0	0.0%	0	0.0%	0	0.0%	3	2.9%	0	0.0%	0	0.0%
	metaplastic	3	5.5%	1	2.0%	0	0.0%	0	0.0%	0	0.0%	0	0.0%
	other	0	0.0%	0	0.0%	0	0.0%	2	2.9%	0	0.0%	0	0.0%
	missing	1		4		0		4		0		1	
T stage	T1	16	30.8%	23	43.4%	1	20.0%	37	43.0%	13	59.1%	12	57.1%
	T2	30	57.7%	26	49.1%	4	80.0%	36	41.9%	8	36.4%	8	38.1%
	T3	4	7.7%	3	5.7%	0	0.0%	12	14.0%	0	0.0%	1	4.8%
	T4	2	3.8%	1	1.9%	0	0.0%	1	1.2%	1	4.5%	0	0.0%
	missing	4		2		1		1		0		1	
nodal involvement	N0	26	50.0%	32	61.5%	2	40.0%	38	43.7%	15	75.0%	13	61.9%
	N+	26	50.0%	20	38.5%	3	60.0%	49	56.3%	5	25.0%	8	38.1%
	missing	4		3		1		0		2		1	

tumor grading	G1	0	0.0%	0	0.0%	0	0.0%	8	9.2%	3	13.6%	0	0.0%
	G2	5	9.3%	9	18.4%	1	16.7%	48	55.2%	5	22.7%	18	81.8%
	G3	49	90.7%	40	81.6%	5	83.3%	31	35.6%	14	63.6%	4	18.2%
	missing	2		6		0		0		0		0	
Ki67 proliferative index	low (≤15%)	1	2.0%	0	0.0%	0	0.0%	30	36.6%	4	20.0%	8	36.4%
	intermediate	8	15.7%	12	25.5%	2	33.3%	35	42.7%	3	15.0%	8	36.4%
	high (>35%)	42	82.4%	35	74.5%	4	66.7%	17	20.7%	13	65.0%	6	27.3%
	missing	5		8		0		5		2		0	
chemotherapy treatment of first occurrence	no	0	0.0%	3	5.5%	0	0.0%	18	20.9%	3	13.6%	10	45.5%
	yes	56	100.0%	52	94.5%	6	100.0%	68	79.1%	19	86.4%	12	54.5%
	missing	0		0		0		1		0		0	
use of platinum compounds in chemotherapy of first occurrence	no (other chemo)	37	67.3%	24	49.0%	2	33.3%	66	100.0%	14	82.4%	11	91.7%
	yes	18	32.7%	25	51.0%	4	66.7%	0	0.0%	3	17.6%	1	8.3%
	n/a - no chemo	0		3		0	0.0%	18		3		10	
	missing	1		3		0		3		2		0	

*Percentages may not total 100 because of rounding; the patient with both gBRCA1m and gBRCA2m was excluded from this overview.
